# Supplementary material for: Are carbon emissions trading and green financial instruments synergistic? -Comprehensive quantitative research based on content analysis
Source: PLoS One. 2024 Mar 7;19(3):e0298601. doi: 10.1371/journal.pone.0298601 (PMC10919601; doi:10.1371/journal.pone.0298601)
Supplement: S1 Data — (ZIP) [file pone.0298601.s001.zip › Data/地级市Z_F工作报告.zip!/┴╔─■/╕o╦│╩╨/└┤╫╘╣1⁄2╓┌║┼í╛┬φ┐╦╩2╛▌═°í┐_2014.docx]

各位代表：

　　现在，我代表市政府向大会报告工作，请予审议，并请政协各位委员提出意见。

　　一、2013年工作回顾

　　2013年是极不平凡的一年。面对严峻复杂的经济形势和前所未有的特大洪涝灾害，在省委、省政府和市委的正确领导下，在市人大、市政协的监督支持下，我们团结带领全市人民，夺取了抗洪救灾的全面胜利和恢复重建的阶段性胜利，保持了经济社会持续平稳健康发展，朝着幸福美丽抚顺迈出了坚实步伐。

　　预计地区生产总值1348亿元、同比增长9%，公共财政预算收入134.2亿元、增长3.1%，全社会固定资产投资1106亿元、增长16%，社会消费品零售总额524亿元、增长15%，城市居民人均可支配收入22600元、增长10%，农民人均纯收入11168元、增长11%。

（一）新城发展提质提速

　　新城活力进一步释放，支撑作用进一步增强。

　　沈抚新城“三区”建设速度加快。产业区装备制造产业集群被确定为国家级产业集群示范基地，投产超亿元项目20个，年销售收入突破700亿元、增长24%；太平洋工业城建设模式全省推广，累计建成标准厂房146万平方米、入驻企业63户。核心区城市功能日渐完善，金风湾、玉露潭成为区内中心公园，“生命之环”成为抚顺新地标；亿丰东北家居中心签约企业507户，总部基地入驻企业50家。生态区巴厘岛水世界、海洋主题乐园再创旅游新高，冰雪欢乐世界投入运营，丰远温泉小镇、辽沈战役景区加快建设。规划、户籍、金融等沈抚同城化取得新进展。

石化新城园区建设和城市更新改造齐头并进。园区基础设施和公用工程建设力度加大，新加坡胜科水务、公共管廊项目投入使用；在建超亿元项目29个、总投资140亿元，汉圣化工、华亿化工等一批重点项目试生产，同益石化、齐隆化工等龙头企业辐射带动作用增强。城区完成安居工程14万平方米、房地产开发30万平方米，以东洲大街和绥化路为轴线的商贸核心区初具形象。

（二）产业结构优化升级

　　坚持以增量调结构、以创新促升级，三次产业实现质量、效益同步增长。

　　工业升级步伐加快。开工建设超亿元项目221个，完成投资710亿元，创历史新高。12个重点工业产业集群完成销售收入1716亿元、增长28.9%，机器人、现代印刷、化工及精细化工、现代焊接四大产业集群取得新进展。深入实施创新驱动战略，完成高新技术产业增加值189亿元、增长15%。抚顺石化、抚矿集团等重点大企业稳增长作用凸显。全年预计完成规上工业增加值597亿元、增长10.5%。

　　服务业质量提升。22个服务业集聚区加快发展。传统服务业取得明显进展，万达广场、绿地商业街、浙商星星国际酒店正式营业。旅游业发展势头强劲，在全省率先成立旅游委，大力实施旅游名城战略，推出6条精品线路，成功举办系列节庆活动，旅游总人数、总收入实现新突破。以电子商务、物流、信息服务为代表的现代服务业取得新进展。“夜经济”成为消费新热点。房地产开发投资和商品房销售额分别增长15%以上。

农业稳步发展。粮食产量65万吨。新增亿元以上农产品深加工企业4户、规模以上产业化龙头企业20户，建成现代畜牧业示范场17个。创建国家万亩高产示范区4万亩，建成万亩中药材谷1个、千亩山野菜园5个、百亩花卉园3个。特色农业占农业总收入52%。

（三）对外开放成效显著

　　以发展定项目，以项目筹资金，对外开放实现新突破。

　　招商引资力度加大。创新招商模式，引进香港长德现代商贸物流城、中国供销东北农商交易城、江苏森茂国际汽车城等一批大项目，全年预计域外引资到位额886亿元、增长25%；直接利用外资5.2亿美元、增长312%，增幅居全省首位。

　　外贸出口势头良好。全年预计外贸出口8.4亿美元、增长19%，超额完成全年外贸出口指标。与沈阳海关签署支持抚顺外向型经济发展和对外开放合作备忘录，抚顺装备制造业基地成功入选第一批省级出口基地。我市外经贸工作获省政府唯一特别奖励。

境外投资实现突破。鼓励支持企业“走出去”，罕王集团投资印尼镍矿产业园被列为中印尼首批镍业重大合作项目，抚矿集团投资约旦页岩油炼化项目进展顺利。海外并购取得新进展，罕王集团成功并购澳大利亚金矿。

（四）城乡环境明显改善

　　统筹城乡发展，突出生态文明建设，人居环境持续改善。

　　大力实施生态工程。我市荣获“国家森林城市”称号，超额完成省下达的“两退一围”、闭坑和生产矿山生态治理任务。建成辽宁东方发电公司等热源管网项目；并网供热面积584万平方米；出租车油改气717台；启动电力、钢铁、水泥、石化行业除尘升级、脱硫脱硝工程；建成PM2.5和重点大气排污企业在线监测系统。5座新建污水处理厂稳定运行，城市污水达标排放；治理城区支流河及农村中小河流12条。

　　完善提升城市功能。新建改造“三供”管网205.6公里。永济路、古城子河立交桥、城东三期“两路两桥”竣工通车。改造城市道路71条，新增绿地95公顷。完成高尔山公园广场和雷锋体育场休闲广场改造。实施小区环境改造和绿化工程30项。启动冬季无公害除雪。更新公交车300台。街路弱电线路下地336条、1911公里，清理乱堆乱建2700处。城区设置垃圾桶2480个、果皮箱4000个。

　　努力建设美丽乡村。积极推进农村环境综合整治，实施村内道路工程591公里、桥梁62座，绿化道路619公里，建设垃圾收集池1181处、污水处理设施12项，改厕3000座，广场建设和边沟治理有序推进，139个行政村达到省级环境标准，4个行政村成为全国美丽乡村创建试点村，全省农村环境治理工作现场会在抚顺召开。

　　全面启动大伙房水源保护区综合治理。坚持上游与下游、公众利益与个人利益、环境保护与经济发展三个统筹兼顾，以确保饮水安全为目标，以促进保护区内经济发展和农民收入明显提高为重点，全面启动保护区综合治理五年计划，省、市、县区成立专门机构，强力推进8大治理工程、88个治理项目。大伙房水源保护区成功列入国家江河湖泊生态环境保护试点，得到国家大力支持。

（五）民生工程扎实推进

坚持民生优先，将更多发展成果惠及全市人民。

社会保障水平逐年提高。按时调增企业离退休人员基本养老金；在全省率先出台困难群体政府贴息借款缴费政策，强化扩面征缴，确保了养老金按时足额发放；提高城镇居民基本医疗保险和新农合政府补贴标准，启动城镇居民大病保险和新农合大病医保工作；被征地农民社会保障制度实现全覆盖；提高城乡低保救助标准和农村五保对象供养标准，惠及16.6万人。实施医疗救助3万人次。妇女小额担保贷款突破5亿元，位居全省第一。加大资金投入，启动社区建设五年规划，提高社区工作者生活补贴。市殡仪馆新馆投入使用。

就业安居力度加大。实名制就业10.5万人，城镇登记失业率2.6%，零就业家庭保持动态为零。拆迁改造旧城区103万平方米，开工建设63万平方米采沉棚户区居民避险搬迁安置工程，建成棚改和保障性住房7943套，安置榆林采沉区和西露天矿北邦居民脱险510户，改造农村危房578户、贫困残疾人危房700户。

教育卫生事业均衡发展。启动15所城区公办高中、中职学校提升改造工程，学前教育三年行动计划顺利完成，新抚、顺城通过省基础教育强区验收，4所高中通过省示范性高中复检，抚顺二中成为清华大学“新百年领军计划优秀学生推荐学校”。加快推进基本公共卫生服务均等化，实施基本药物制度；改善就医环境，市中心医院病房楼、市中医院新楼主体完工；引进优质医疗资源，市中心医院和第二医院分别与中国医大附属一院和盛京医院开展深度合作。免费孕前优生健康检查目标人群覆盖率100%。

文化体育事业加快发展。市图书馆新馆主体竣工，市群众艺术馆、朝鲜族文化馆开工建设，农民健身工程覆盖率90%。圆满承办“十二运”公开水域赛事，成功举办群众文化节、全民健身节。

平安抚顺建设积极推进。公共安全形势持续稳定，无重大级别以上安全生产事故，刑事发案率下降16%。化解个人、集体访974件。首个“留守儿童法律援助工作站”挂牌成立。

（六）抗洪救灾与恢复重建取得胜利

　　“8·16”特大洪灾破坏之大、损失之重前所未有，造成重大人员伤亡和财产损失。面对突如其来的洪灾，我们有力、有序、有效应对，广大党员干部尽职尽责、率先垂范，解放军、武警消防官兵、公安干警舍生忘死、连续奋战，社会各界心系灾区、无私援助，受灾群众自力更生、抗灾自救。全市紧急转移安置群众16.3万人，争取上级资金13.67亿元，自筹资金2.18亿元，接受社会捐助9883万元，在最短时间内恢复了灾区交通、电力、通信等基础设施，以最快速度启动了灾后恢复重建，保证了大灾之后无疫情、受灾群众生活有保障，灾区社会和谐稳定，经济持续平稳发展。灾害面前，我们心手相连，守望相助，共同谱写了众志成城、抗灾自救、重建家园的恢弘篇章。

（七）政府自身建设不断加强

把建设人民满意政府作为不懈追求，政府执行力和公信力进一步提升。

　　自觉接受市人大及其常委会的法律监督、工作监督和市政协的民主监督。办理人大代表建议375件、政协委员提案427件，办结率100%、满意率99.8%，综合解决率比上年提高2个百分点。办理市长公开电话3.8万件次，办结率100%、群众满意率98.7%。

　　强化作风建设，贯彻落实中央八项规定和反对“四风”要求，开短会、发短文，深入基层，走进项目，在发展一线破解难题。坚持真抓实干，重信守诺，对年初确定的目标任务，明确责任，强化督查，说到做到，做就做好。

　　推进廉洁型、服务型政府建设，理顺工程招投标、政府采购、产权交易管理体系，组建专门管理机构，规范公共资源交易市场秩序，完成交易项目1123项，实现交易额41亿元。

　　牢固树立过紧日子思想，厉行节约，反对铺张浪费，“三公”支出减少23%。

　　其它各项工作都取得了新进展。

　　各位代表，一年来，我们真抓实干，开拓进取，实现了有质量的稳定增长、可持续的全面发展。成绩的取得，是省委、省政府和市委正确领导的结果，是市人大、市政协监督支持的结果，是广大党员干部扎实工作的结果，是全市人民团结奋斗的结果。在此，我代表市政府，向全市各族人民，向人大代表、政协委员，向各民主党派、工商联、无党派人士、人民团体、离退休老同志，向驻抚部队、武警官兵、公安干警和中省直单位，向所有关心抚顺发展的朋友们，表示崇高的敬意和衷心的感谢！

　　看到成绩的同时，我们也清醒地认识到面临的诸多困难和问题。一是经济结构不尽合理。服务业比重低，民营经济不发达，大企业大项目少，金融对经济的支持作用不够。二是历史包袱重。人口老龄化程度加剧，社会保障压力逐年加大；城市基础设施老旧，旧城区改造任务依然艰巨。三是创新社会管理，解决好群众关心的上学、看病、就业、出行、供暖等民生问题，还需要付出更多的努力，部分群众生活还很困难。四是节能减排和生态环境保护形势依然严峻，空气质量亟待改善。五是机关作风、工作效率与群众期待、发展需要还有差距。对于这些问题，我们将采取有力措施，认真加以解决。

二、2014年主要工作

2014年是实施城市转型的第一年。全市经济社会发展的总体思路是：以党的十八届三中全会精神为指导，全面贯彻落实市委全会暨经济工作会议精神，坚持稳中求进、进中求好的工作总基调，以项目建设为牵动，以改革开放为突破口，深入实施“两城两带”、全域发展、多点支撑的发展战略，全力推进城市转型，加快建设幸福美丽抚顺。

　　全市经济社会发展的预期目标是：地区生产总值增长9.5%，公共财政预算收入增长10%，全社会固定资产投资增长20%，社会消费品零售总额增长14%，城市居民人均可支配收入增长12%，农民人均纯收入增长12%。

　　实现上述目标，要着力抓好以下十项工作：

（一） 全面启动消费主导型城市建设

建设消费主导型城市是一个长期、渐进的过程，今年必须全面启动。

　　明确目标。以全面扩大和提升城市消费需求为主线，大力培育和发展生活性、生产性和发展性消费，努力构建具有抚顺特色的消费框架体系，将抚顺建设成为沈阳消费产业群的重要部分。

　　制定方案。强化顶层设计，编制经济、社会、体制、功能和文化等全面转型的总体方案，制定新城、县区等区域转型和工业、服务业、农业等产业转型以及公共服务转型的具体方案。

启动规划。按照统筹兼顾、远近结合的原则，以城市景区化、景区城市化、产城一体化的发展理念，高标准编制总体规划和专项规划，以规划引领转型。

出台政策。制定扶持消费产业发展、鼓励本地消费和吸引外来消费政策，推出鼓励民间投资和扩大融资政策，以政策资金的支持，推动城市转型。

（二）全力推进新城和开发区建设

改革区域管理体制，构筑产业升级新高地、城市转型先导区。

沈抚新城围绕打造全国最具影响力的机器人和现代印刷产业集群，建设产学研一体、上下游配套、服务平台完备的产业基地，加快罕王微电子产业园建设，确保百级传感器芯片和类人智能机器人产品顺利投产。全年新开工超亿元项目100个，太平洋工业城新建标准厂房100万平方米、入驻企业50户。加快德商凤巢酒店、君悦豪生酒店建设，建成运营城堡酒店、欧洲风情商业街、生态科普世界，支持沈阳名校设立分校。完善城市功能，建设沈抚综合客运枢纽站，市中医院新楼投入使用。进一步加快沈抚同城化步伐。

石化新城围绕打造国家级化工及精细化工产业基地，充分发挥资源优势，着力延伸产业链，引进世界500强霍尼威尔催化剂、华兴石化石油树脂重点项目，确保佳化化学等在建项目投产达效。加快园区基础设施和公用工程建设，中电投热电联产、营口港陆港项目开工建设。启动新城路、临江路东延工程，甲邦大桥建成通车。建设河滩公园，形成新的水上景观带。

望花开发区和胜利开发区加快建设园区基础设施和牵动性项目，明晰主导产业，形成特色鲜明的产业区，全力打造南环产业带。

（三）全面提升工业竞争力

坚持增量与存量并重，增强工业支撑作用。规模以上工业增加值增长11%。

　　做大增量。大力发展12个重点工业产业集群，积极推进工业地产建设，力争机器人、现代印刷、化工及精细化工、现代焊接四大产业集群取得明显进展。全年实现销售收入2100亿元、增长20%，新增工业地产180万平方米以上。

　　做强存量。加快石化、冶金、装备制造等传统优势产业发展，三大产业实现产值2110亿元、增长12.3%。积极推进22个省级重大工业、新兴产业和重点科技项目建设，抓好173个超亿元工业技改项目，推动挖掘机、水泥厂、新钢铁搬迁改造。全年工业投资完成820亿元。

做优环境。出台鼓励政策，活跃民间投资，探索发展混合所有制经济。构建科技创新体系和多元化融资体系，促进中小微企业发展。全年民营经济实现增加值954亿元，增长13%。

（四）加快发展服务业

　　坚持传统服务业与现代服务业融合发展，促进服务业发展提速、比重提高、水平提升。

　　促进传统服务业升级。鼓励原有大型商贸企业多元化发展，推进恒大国际广场、兴隆摩尔世界等城市综合体建设。积极推进工业企业主辅分离，促进生产性服务业发展。活跃夜间消费市场。以中国供销东北农商交易城为牵动，建设榆林市场群，将榆林采沉区打造成国家资源型城市可持续发展示范区。

　　提升旅游产业水平。深入实施旅游名城战略，确立大旅游、大产业发展理念，加强区域合作，扩大对外宣传，打造知名品牌。强化项目带动，提档升级一批、建成运营一批、开工建设一批、策划推进一批重点旅游项目，做好冰雪、温泉、乡村、沟域旅游招商，办好节庆活动，形成旅游品牌。全年旅游总收入增长20%。

　　大力发展现代服务业。加快发展物流、动漫、金融、电子商务、文化创意等现代服务业。启动香港长德现代商贸物流城、东方龙动漫产业园、金融生态城市和东北网贸港网商基地建设；亿丰电子商务中心完成交易平台建设；鼓励扶持文化产业发展壮大，建设新抚文博产业城和沈抚新城出版创意产业园。

　　积极发展房地产业。加大政策扶持力度，采取有力措施，促进房地产市场持续稳定健康发展。全年拆迁改造旧城区100万平方米，房地产开发投资和商品房销售额分别增长15%以上。

（五）大力发展县域经济

以富民强县为目标，提高县域经济发展质量和农民收入水平。

　　加快推进农业产业化。落实科技惠农政策，优化农业产业结构，稳定粮食生产。以大伙房水源保护区综合治理为契机，调优种植业和养殖业结构，大力发展高效农业、设施农业、生态农业和旅游观光农业，做大食用菌、山野菜、中药材和百合花特色产业基地规模，建立现代农业产业体系。启动百万亩省级有机农业生产示范基地建设，发展有机农业产业基地10万亩。大力发展生态畜牧业和农民专业合作社。

　　加快推进县域工业化。用好省支持水源保护政策资金，加快园区基础设施建设和龙头企业引进。重点抓好新宾县现代焊接、清原县输变电、抚顺县炭化木等工业产业集群。全年县域工业产业集群实现销售收入202亿元，增长26.3%。

加快推进农村城镇化。遵循规律，因势利导，多渠道筹措资金，积极稳妥推进城镇化。清原县加快推进铁北地区配套基础设施和南口前新市镇建设，新宾县加快推进西部新城建设，抚顺县加快推进石文新市镇和后安中心镇建设，提高人口集聚能力。

　　（六）扩大对外开放

　　进一步加大对外开放力度，推动抚顺在更高层次上发展。

　　强化招商引资。着力引进一批大企业、大项目，引进一批科研院所、研发中心，引进一批国内外高端人才。域外引资到位额1045亿元、增长18%，直接利用外资5.8亿美元、增长12%。

　　促进外贸出口。加强出口基地建设，支持具有自主品牌的产品出口，提高外贸依存度。鼓励企业境外投资。全年外贸出口9.4亿美元，增长12%。

　　推动项目建设。完善重大项目协调推进机制，加快清原抽水蓄能电站、六星汽车文化产业园等重大项目落地，提高招商项目合同履约率、资金到位率、项目开工率。全年开工建设超亿元项目470个，完成投资1050亿元。

强化要素保障。坚持节约集约用地，优先保障成熟项目和重点项目用地。扩大融资渠道，支持企业通过资本市场直接融资，鼓励民间资本进入公用事业领域，做好辽宁第一期小微企业增信集合债券试点工作。强化政府债务管控，防范债务风险。全年新增贷款100亿元、增长25%，非信贷融资120亿元，向上争取资金力争突破100亿元。

（七）下大力气建设美丽抚顺

　　继续保持百亿元以上城建投资规模，更加自觉地保护生态，更加积极地改善环境。

　　加强生态保护。深入推进青山工程，实施大伙房水源一级保护区退耕还林还草计划和浑河、苏子河、社河干支流生态封育计划，完成荒山造林7.4万亩、经济林产业带16.3万亩。继续实施绿水工程，统筹建设乡镇污水处理设施，综合治理浑河干支流，基本建成大伙房水库上游干支流生态带。全力开展蓝天工程，坚决控制建设、交通扬尘，搬迁重污染企业，下决心整治龚家沟环境污染问题，推广出租车、公交车油改气，加快供暖“拆小并大”和生活垃圾焚烧厂项目建设，实现电力、冶金、水泥、石化行业污染物达标排放，确保空气质量明显改善。

　　建设宜居城市。完善城市功能，启动辽宁中环高速公路抚顺段建设，规划建设中部通道和北环通道，加快推进新华桥重建，规划新建贵城大桥、安城街大桥和抚挖立交桥，形成东西畅通、南北互联的交通网络，实现城市北拓，使高尔山成为城中山。继续实施城区街巷路改造、小街小巷路灯安装、老旧房屋暖房子工程。新建燃气管网30公里。引水入城，建设水清、岸绿、景美的水城。实行全方位、立体化绿化，启动国家园林城市建设。充分发挥月牙岛生态公园的辐射带动作用，加快华山地区改造。推进城东三期开发，规划建设浑河滩地公园，打造功能齐全的宜居新区；加快推进榆林采沉区生态湿地公园建设。提高城市精细化管理水平。

　　建设美丽乡村。统筹规划，因地制宜，加快重点乡镇、村屯基础设施和生态环境建设。加强农村环境综合整治，推进农村道路两侧、河流两岸“三堆”治理工作，加快农村垃圾处理和无害化厕所建设。维修县级以上公路400公里，改造农村公路160公里，建设村内道路340公里。实施村屯绿化项目120个，达到省级绿化示范村标准。

　　我们深知，环境是最稀缺的资源，生态是最宝贵的财富，绿水青山就是金山银山。我们要以更大的决心和气魄，保护生态，治理环境，让抚顺的天更蓝、地更绿、水更净、空气更清新！

　　（八）切实保障和改善民生

　　提升社会保障水平。强化企业养老保险扩面征缴，确保企业离退休人员养老金按时足额发放。鼓励支持创业，增加居民财产性和经营性收入，切实提高居民收入水平；实施积极的就业政策，全年城镇实名制就业9.5万人。继续加快棚户区、城乡危旧房改造和廉租房、保障性住房建设步伐，改善城乡居民居住条件。完善社会救助体系，适时提高城乡低保救助、农村五保供养标准。完善医疗救助政策，实施城镇居民大病保险，提高参保人员保障水平，解决城乡困难群众看病难问题。完善农村养老试点工作，提高公办养老机构保障能力，积极发展养老服务产业。继续发放妇女小额担保贷款，扶持农村家庭脱贫致富。

　　提升公共服务水平。办好人民满意教育，推进基本公共教育服务均等化，提高义务教育、高中教育优质均衡发展水平，加快职业教育创新发展，培养一流校长和教师队伍，提升各级各类教育质量。办好人民满意医疗，提高新农合筹资和大病保险水平，扩大基本药物制度实施范围，改善就医环境、诊疗技术和服务水平，改造市妇幼保健院。制定出台政策，留住、吸引人才。加快文化体育设施建设，建成全市公共电子阅览室，搬迁市图书馆，筹建体育中心；实施文化体育惠民工程，开展群众广泛参与的文体活动；加强文物保护工作，兴建元帅林石刻博物馆。建设残疾人综合服务中心，投入使用老年活动中心、妇女儿童活动中心、青少年宫。加强食品药品监管，确保群众吃上安全食品、用上放心药品。更新公交车辆，加快场站建设，提高服务水平，有效解决群众出行难问题。加快建设农村安全饮水工程。

　　提升社会管理水平。落实安全生产责任制，完善防灾减灾体系，提高应急处置能力，提升灾害防御水平，切实保障人民群众生命财产安全。建设智慧抚顺，建成城市地下综合管网、气象、卫生、教育、民政等信息平台和交通治安高清视频监控系统。建设平安抚顺，加大社会治安防控力度。改善社区办公条件，新建和改扩建城市社区服务站35个，完成居委会换届选举。畅通诉求渠道，强化法律援助，维护群众合法权益。规范物业企业管理行为，提高物业服务质量，全市住宅物业管理覆盖率达到80%。加强汽车检测线建设与管理，解决检车难问题。支持驻抚部队建设，争创全国“双拥”模范城。

　　抓好灾后重建工作。加强灾后重建组织领导，强化对口帮扶，落实保障资金，严格按照时限完成重建任务，确保家家有房住、人人有保障、设施有提高、经济有发展、生态有改善。

各位代表，群众利益无小事，民生问题大于天，人民政府为人民。我们将想群众之所想、急群众之所急、干群众之所需，让人民生活每年都有新变化，一年更比一年好！

（九）努力建设人民满意政府

　　加强依法行政。严格依照法定权限行使权力，自觉接受市人大及其常委会的法律监督、工作监督和市政协的民主监督，充分听取民主党派、工商联、无党派人士和各人民团体的意见和建议，广泛接受新闻舆论和社会公众监督，让人民监督权力，让权力在阳光下运行，做到干部清正、政府清廉、政治清明。

　　转变政府职能。全面清理和削减行政审批事项，承接好中央和省下放地方的审批权限，激发市场主体和社会创造能力，增强经济社会发展内生动力，提升公共服务水平，维护社会公平正义，打造优良的发展软环境。

　　改进工作作风。认真开展党的群众路线教育实践活动，强化行政问责、审计监督和效能监察，突出整治庸懒散奢等不良风气。大兴求真务实之风，干实事，敢作为，勇担当，以我们的“勤奋指数”提升城市的“发展指数”，以我们的“辛苦指数”提升群众的“幸福指数”。

坚持民本为先。时刻换位思考，关切群众感受，设身处地为群众排忧解难，办好事关人民群众切身利益的民生实事，解决好人民群众反映强烈的突出问题，以实际行动取信人民。

（十）积极稳妥推进各项改革

　　根据省委、省政府和市委的统一部署，全面深化改革，立行不拖延，率先不抢跑，破除一切妨碍科学发展的体制机制弊端，促进经济社会全面转型。

　　遵循顶层设计抓落实。对于涉及面广、需要国家决策部署的重大改革，提前做好工作准备，积极开展政策对接，全面抓好贯彻落实，确保改革措施落到实处、取得实效。

　　围绕既定任务抓推进。对于教育、人口、卫生、文化管理体制、投融资体制、城市管理体制、国有资产管理体制、食品药品安全监管体制等方向明确、路径清晰的改革，要全力推进，尽快突破。

　　突出重点领域抓深化。深化国企改革，推进混合所有制经济快速发展；深化水、电、燃气等资源性价格改革，推行阶梯价格；促进农村土地有序流转，推进土地集约化经营。围绕市场准入、市场监管、公平竞争等关键环节进行综合配套改革，使各项改革措施协同配合、良性互动，为发展探索新途径、提供新动力。

　　各位代表，抚顺正处在转型发展、全面振兴的关键时期。我们重任在肩，使命光荣。让我们在市委的坚强领导下，在市人大和市政协的监督支持下，解放思想，改革创新，奋力拼搏，为加快城市转型、建设幸福美丽抚顺而努力奋斗！
